# Characterization of Glycosyltransferase Family 1 (GT1) and Their Potential Roles in Anthocyanin Biosynthesis in Maize

**DOI:** 10.3390/genes14112099

**Published:** 2023-11-18

**Authors:** Huangai Li, Yiping Li, Xiaofang Wang, Ziwei Jiao, Wei Zhang, Yan Long

**Affiliations:** 1 Research Institute of Biology and Agriculture, Shunde Innovation School, University of Science and Technology Beijing, Beijing 100083, China; huangaili@ustb.edu.cn (H.L.); m202110886@xs.ustb.edu.cn (Y.L.); b20200410@xs.ustb.edu.cn (X.W.); 2 Industry Research Institute of Biotechnology Breeding, Yili Normal University, Yining 835000, China; jiaziwei123@163.com (Z.J.); zhangw891@nenu.edu.cn (W.Z.); 3 Beijing Engineering Laboratory of Main Crop Bio-Tech Breeding, Beijing International Science and Technology Cooperation Base of Bio-Tech Breeding, Zhongzhi International Institute of Agricultural Biosciences, Beijing 100192, China

**Keywords:** glycosylation, glycosyltransferase, GT1, phylogenetic analysis, gene duplication, molecular docking, anthocyanin biosynthesis

## Abstract

Glycosyltransferase family 1 (GT1) is a large group of proteins that play critical roles in secondary metabolite biosynthesis in plants. However, the GT1 family is not well studied in maize. In this study, 107 *GT1* unigenes were identified in the maize reference genome and classified into 16 groups according to their phylogenetic relationship. *GT1s* are unevenly distributed across all ten maize chromosomes, occurring as gene clusters in some chromosomes. Collinearity analysis revealed that gene duplication events, whole-genome or segmental duplication, and tandem duplication occurred at a similar frequency, indicating that both types of gene duplication play notable roles in the expansion of the *GT1* gene family. Expression analysis showed *GT1s* expressing in all tissues with specific expression patterns of each *GT1*, suggesting that they might participate in multiple biological processes during the whole growth and development stages. Furthermore, 16 *GT1s* were identified to have similar expression patterns to those of *anthocyanidin synthase* (*ANS*), the critical enzyme in anthocyanin biosynthesis. Molecular docking was carried out to examine the affinity of GT1s with substrates in anthocyanin biosynthesis. This study provides valuable information on the *GT1s* of maize and will promote the development of research on their biological functions in the biosynthesis of other secondary metabolites.

## 1. Introduction

Glycosylation is one of the most abundant and significant modifications in plant cells [1], which plays a vital role in multiple biological processes like plant growth [2], the biosynthesis of a significant amount of secondary metabolites [3,4,5,6], the precise regulation of the contents of various hormones [7,8,9,10,11], and the defense response to biotic/abiotic stresses [12,13,14]. Glycosylation reactions are mainly catalyzed by glycosyltransferases (GTs, EC 2.4.x.y), which mediate the transfer of sugar moieties from activated donors onto various acceptors, for example, sugars, proteins, nucleic acids, antibiotics, lipids, and other small chemical molecules, to form glycosidic bonds [15,16]. In plants, uridine diphosphate (UDP)-glucose (UDP-Glc) is the most favored sugar donor in glycosylation reactions. In addition, UDP-arabinose (UDP-Ara), UDP-galactose (UDP-Gal), UDP-glucuronic acid (UDP-GlcUA), UDP-rhamnose (UDP-Rha), and UDP-xylose (UDP-Xyl) can also serve as glycosyl donors for GTs [5,17]. According to the different types of glycosidic bonds formed, GTs can be divided into O-, S-, N-, and C-glycosyltransferases, with O-glycosides being the most widely distributed and well-known glycosylation products [18].

Glycosyltransferases available from the Carbohydrate-Active enZyme (CAZy, http://www.cazy.org/, accessed on 3 September 2023) database are classified into 116 families according to protein sequence similarity, the stereochemical structure of glycosidic bonds, and substrate specificity, and the vast majority of GTs belong to the family 1 (GT1). The common sugar donor of GT1s is UDP-Glc; thus, they are also called UGTs [19]. In *Arabidopsis*, about 120 genes were predicted to encode GT1s, which were phylogenetically classified into 14 distinct groups [20]. Most of the GTs are reported to be associated with the biosynthesis of secondary metabolites of lignin and flavonoid, which fall into the GT1 family [21,22,23]. Some GT1s, referred to as UDP flavonoid glycosyltransferase (UFGT) or 3-O-glucosyltransferase (3GT), catalyze the last step in the biosynthesis of anthocyanins, and anthocyanidin synthase (ANS) mediates the penultimate processing step. The glycosylation process of anthocyanins can improve the solubility and stability of anthocyanins in plants [24]. Due to the importance of secondary metabolites in biology, pharmacology, and agronomy, researchers have continuously focused on the study of GT1s in recent decades.

The structural information of GT1s is of great significance for discovering the glycosylation catalytic mechanisms. Most plant GT1s have a conserved motif consisting of 44 amino acid residues at their C-terminus, namely the plant secondary product glycosyltransferase (PSPG) box, which is believed to function in the binding of glycosyl donors [3]. Except for the PSPG box, GTs have relatively low sequence similarity and the N-terminal is significantly variable among sequences, suggesting their diversity in the substrate binding of receptors [16].

Maize is an important and widely distributed cereal crop. It serves as human food, livestock feed, biofuel, and raw industrial materials. Based on the universal uses, it is worth characterizing maize GT1s and better understanding their functions in critical biological processes. Bioinformatics has been widely used to characterize gene families in maize and other plant species [20,25,26,27,28,29,30]. In the present study, we identified 107 *GT1* members, which were then subjected to a series of bioinformatics analyses to show their phylogenetic relationship, chromosomal location, conserved motifs and domains, gene structure, and gene duplication events of GT1s in the whole genome as well as expression profiles to predict the candidate genes involved in the biosynthesis of anthocyanin. Molecular docking analysis was further conducted to test the affinities of GT1s with the substrates. This study may provide a comprehensive insight into characterizing the maize GT1s, thus promoting the functional elucidation of GT1s in the biosynthesis of important chemicals of interest.

## 2. Materials and Methods

### 2.1. Identification of Maize GT1s

The GT1 proteins of maize with accession numbers were retrieved from the CAZy Database (http://www.cazy.org/, accessed on 3 September 2023) [31]. The corresponding protein sequences were obtained from the National Center for Biotechnology Information (NCBI, https://www.ncbi.nlm.nih.gov, accessed on 5 September 2023) and blasted against the amino acid sequences of the maize reference genome (Zm-B73-REFERENCE-NAM-5.0) [32] deposited on EnsemblPlants Database (https://plants.ensembl.org/index.html, accessed on 5 September 2023). Redundant sequences were removed manually.

### 2.2. Multiple Sequence Alignment of GT1s

The alignments of GT1 protein sequences were performed using ClustalW program implemented in MEGA11, and then visualized graphically using ESPript 3.0 (https://espript.ibcp.fr/ESPript/cgi-bin/ESPript.cgi, accessed on 12 September 2023) [33].

### 2.3. Phylogenetic Analysis of GT1s

The *Arabidopsis* GT1 protein sequences were downloaded from the EnsemblPlants Database (https://plants.ensembl.org/index.html, accessed on 13 September 2023). The phylogenetic trees of GT1 proteins from maize and *Arabidopsis* were constructed according to the maximum likelihood (ML) method using One Step Build a ML Tree program implemented in TBtools v2.003 [34]. The topology of each phylogenetic tree was assessed through a bootstrap resampling analysis with 5000 replicates. The tree was visualized and modified using the online Evolview tool (http://www.evolgenius.info/evolview/, accessed on 13 September 2023) [35]. All GT1s were classified based on their phylogenetic relationship with GT1s previously identified in *Arabidopsis* and maize [25,36].

### 2.4. Gene Structure and Conserved Motif Analysis

Prediction of conserved motif was performed using the MEME (http://meme-suite.org/, accessed on 13 September 2023) with the parameter of number of unique motifs = 10. Conserved domains were identified using the Web CD-Search Tool on NCBI (https://www.ncbi.nlm.nih.gov/Structure/bwrpsb/bwrpsb.cgi, accessed on 13 September 2023). Gene structures were predicted according to the gene annotation file downloaded from EnsemblPlants Database (https://plants.ensembl.org/index.html, accessed on 2 August 2023). Finally, the phylogenetic analysis, conserved motifs and domains, and gene structure analysis were merged using TBtools [34].

### 2.5. Chromosomal Localization and Collinearity Analysis for Duplicated Genes

All the *GT1* genes were mapped on maize chromosomes and visualized using TBtools [34] according to their physical positions in the annotation file from EnsemblPlants (https://plants.ensembl.org/index.html, accessed on 2 August 2023). The collinearity analysis was carried out using the One Step MCScan Program in TBtools (e-value ≤  1 × 10^−10^) to identify the tandem, whole-genome duplications (WGDs), or segmental duplicated genes. The WGD or segmental duplicated *GT1* genes were further marked on the genomes using Advanced Circos program of TBtools [34].

### 2.6. Expression Profile Analysis

The gene expression patterns were measured using the RNA-sequencing data published previously [37], which were downloaded from the qTeller platform of MaizeGDB (https://qteller.maizegdb.org, accessed on 16 Septermber 2023). Subsequently, the heatmap of *GT1* expression was generated using TBtools [34].

### 2.7. Molecular Docking of GT1s

The structural formulae (SDF format) of UDP-Glc, pelargonidin, cyanidin, and delphinidin were downloaded from the PubChem database (https://pubchem.ncbi.nlm.nih.gov, accessed on 19 July 2023), and subsequently imported into Chem3D software (v18.0) for optimization and energy minimization using the MM2 module. The energy-minimized molecules were further served as ligand input during the docking simulation. The crystal structures of GT1 receptors were obtained from the MaizeGDB database (https://maizegdb.org/, accessed on 11 July 2023) and processed using Pymol v2.5.5 to remove all small and ligand molecules. Next, the PDBQT files for the receptors and ligands were generated using AutoDockTools v1.5.6 [38]. The GT1 receptors were processed by adding hydrogen and charges, and the ligands were prepared by adding atomic charges and assigning atom types. The docking pocket was determined using AutoGrid [38], and molecular docking was carried out using AutoDock Vina [39,40] to search for the best docked conformation. The conformations with the lowest binding energy were selected to analyze the interactions between receptor and ligand. The docking simulations were visualized using LigPlot (2D) [41].

## 3. Results

### 3.1. Identification and Phylogenetic Analysis of Maize GT1s

A total of 316 GT1 protein sequences of different maize lines were collected from the CAZy database [31], 145 of which were isolated from inbred line B73; 29 were from 12 other lines including McC, W22, Mo17, I137TN, Coroico, CML258, NalTel, RP4Htn1, Ngo dim Dak Lak, CI 31 A, maxicana, and A188; and the other 142 were submitted without source information. Through sequence similarity searching in the maize reference genome (Zm-B73-REFERENCE-NAM-5.0) [32], 107 unigenes were identified as encoding the above GT1 proteins (Table 1). The 107 GT1s encoded products with a variable length between 427 and 525 amino acids (average 480 amino acids) and each proved to contain the conserved PSPG motif (Table 1 and Figure S1).

**Figure 1:**
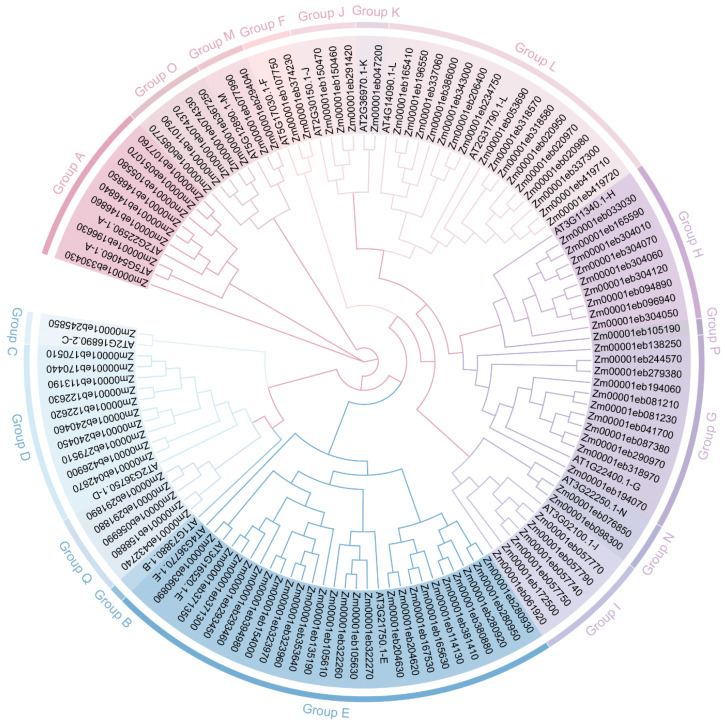
Phylogenetic analysis of GT1 family members from maize and *Arabidopsis*. Phylogenetic tree of *GT1* genes constructed using the maximum likelihood (ML) method using TBtools. The Bootstrap value was 5000 replicates. The colored background represents the different groups of GT1s.

To assess the evolutionary relationship between GT1s, we conducted a phylogenetic tree of maize GT1s. The 107 GT1s were clearly categorized into 16 groups (Groups A, C–Q), consistent with the GT1 phylogenetic classification established previously in *Arabidopsis* and maize [25,36] (Figure 1). Group E comprised the most GT1 members (*n* = 25), followed by Group L (*n* = 17) and Group G (*n* = 11), while Groups C, K, and P each contained only one member. However, no GT1s identified in this study were classified as members of Group B.

### 3.2. Motif Composition, Conserved Domain, and Gene Structure Analyses of GT1 Genes

**Figure 2:**
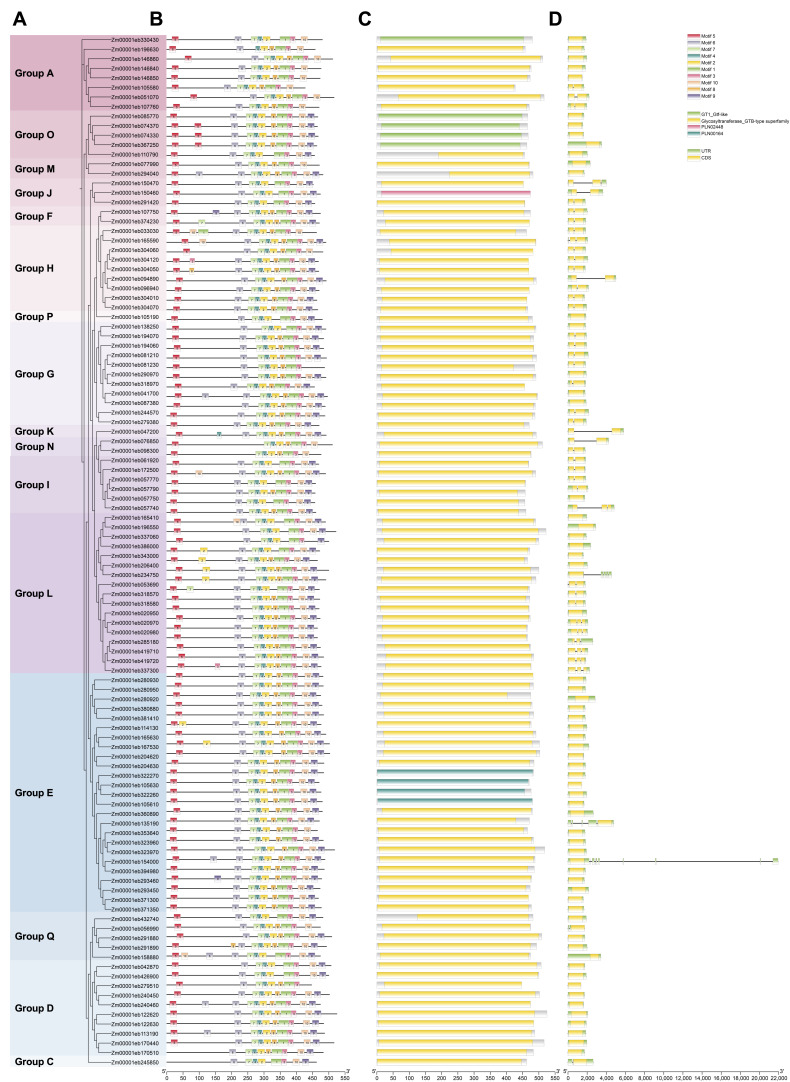
Conserved motifs, domains, and gene structures of maize GT1s. (**A**) Phylogenetic relationship of GT1 members. (**B**) Motif compositions of GT1s. Ten different motifs are shown using variously colored boxes. (**C**) Conserved domains were predicted using MEME. (**D**) Gene structure of GT1s.

To better understand the structural features of GT1 proteins, the phylogenetic tree, motif identification, conserved domains, and gene structure analyses of 107 GT1s were merged together. The phylogenetic relationship of GT1s of maize only was consistent with that constructed together with GT1s of *Arabidopsis* (Figure 1 and Figure 2A). We further examined 10 highly conserved motifs within each GT1 using the MEME tool. The results showed that most GT1 proteins of the same group exhibited similar motif compositions, suggesting functional similarities in the glycosyltransferase family. The number of motifs in each protein ranged from 8 to 11. About 65% (*n* = 70) of GT1 members contained all ten motifs, while the others (*n* = 30) lacked one or two (Figure 2B). The conserved PSPG motif sequence was detected in motif 1, which was present in all GT1 proteins (Figure 2B and Table S1). In addition, we also found the presence of duplication of the motifs in some GT1 members, 26 of which had one duplicated motif and 2 contained two duplicated motifs (Figure 2B). Furthermore, the conserved domains of GT1 proteins were also investigated. As expected, all of the GT1 proteins harbored glycosyltransferase-related conserved domains, including the Glycosyltransferase_GTB-type superfamily, GT1-Gtf-like, PLN02448, and PLN00164 (Figure 2C).

To analyze the gene structure of the *GT1s*, we examined the composition of introns and exons using the annotation file. Over 97.2% of *GT1* family members (*n* = 104) contained a small number of introns. Among these *GT1* genes, 61 *GT1s* had no introns, 34 had only one intron, and 9 had two introns (Table 1). Most of the introns in these *GT1s* were located within coding sequence (CDS) regions but rarely in untranslated regions (UTRs). However, *Zm00001eb135190*, *Zm00001eb154000,* and *Zm00001eb234750* had more introns with 4, 9, and 4, respectively, and these introns distributed within 5′ and 3′ UTRs (Figure 2D and Table 1). The structural divergences in *GT1s* may lead to functional differentiation in substrate identification and enzyme activity.

### 3.3. Chromosomal Distribution and Gene Duplication of GT1 Genes

**Figure 3:**
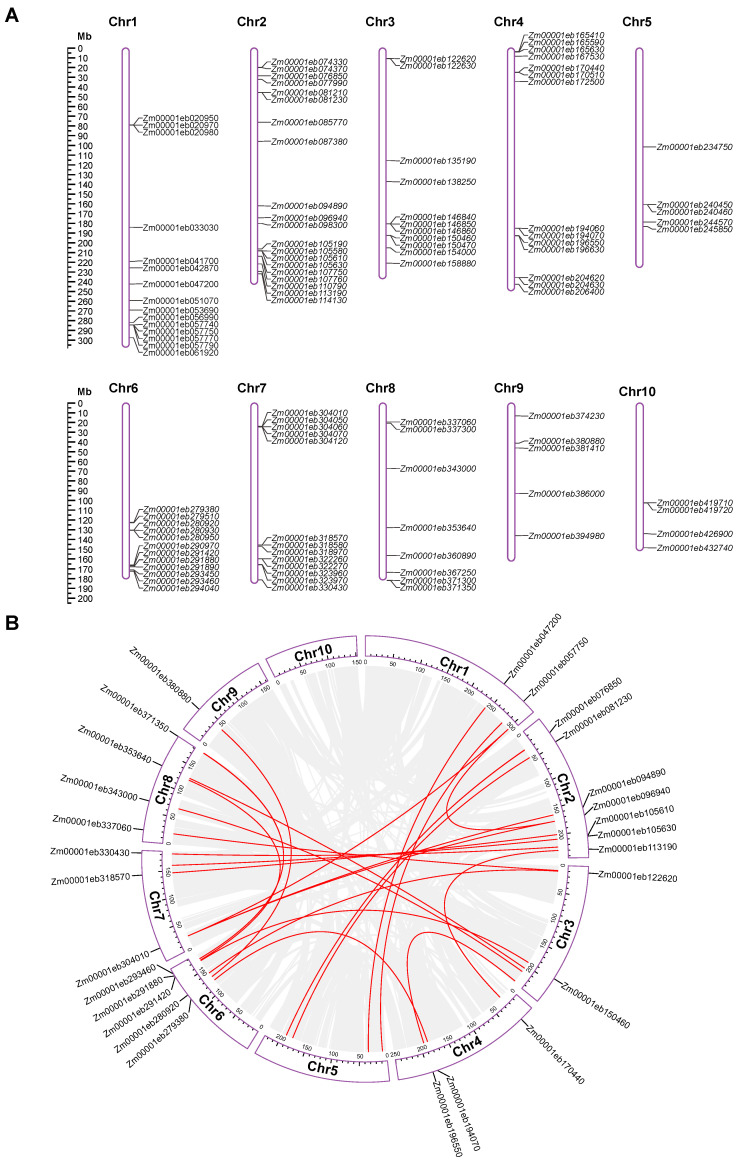
Chromosomal distribution and collinearity analysis of maize GT1s. (**A**) Distribution of GT1 gene family on ten maize chromosomes. (**B**) Collinearity analysis of maize GT1s. Gray lines indicate syntenic blocks within the maize genome, and red lines represent duplicated GT1 gene pairs.

All of the *GT1* genes were mapped onto the ten maize chromosomes to visualize their chromosomal distribution. The *GT1* genes were unevenly distributed across all ten maize chromosomes and chromosomes 2 and 10 contained the highest (*n* = 20) and lowest (*n* = 4) numbers, respectively (Figure 3A and Table 1). Interestingly, *GT1* genes often tended to form small gene clusters on the chromosomes (Figure 3A). Based on the chromosomal distribution, we identified 25 *GT1* gene clusters, each harboring two or more *GT1* gene family members. The numbers of *GT1* gene clusters on each chromosome were as follows: 2 on Chr1, 4 on Chr2, 3 on Chr3, 5 on Chr4, 1 on Chr5, 4 on Chr6, 4 on Chr7, 1 on Chr8, 0 on Chr9, and 1 on Chr10. In general, *GT1* genes in the same cluster fell into the same phylogenetic group, with few exceptions, such as *GT1* clusters 4, 8, and 10 (Table S2).

Gene duplications of the 107 *GT1* genes were investigated using MCScanX and TBtools. The analysis results revealed that 25 gene pairs from 29 *GT1* genes, belonging to 11 groups including A, D, E, G-L, N, and Q, appeared to have arisen from WGD or segmental duplications (Figure 3B, Table 1 and Table S3). These gene pairs were distributed on nine maize chromosomes and most frequently on Chr2, similar to the *GT1* distribution (Figure 3). Meanwhile, 29 *GT1* genes were likely to be tandem duplicates (Table 1), suggesting that WSD or segmental duplication and tandem duplication played comparably important roles in the evolution of the *GT1* gene family.

### 3.4. Expression Analysis of GT1 Genes

**Figure 4:**
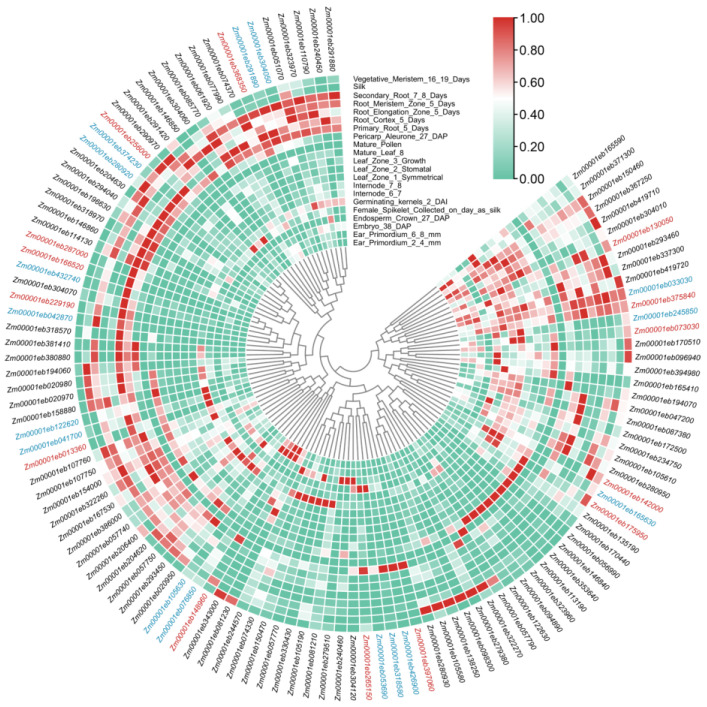
Heat map of *GT1* and *ANS* gene expression in multiple maize plant tissues. The relative expression levels are depicted according to the color scale, where a change from green to red indicates transcript abundance from low to high. The phylogenetic relationships are shown in the center. Gene IDs in red represent the *ANS* genes and those in blue represent the selected *GT1s* clustered together with *ANSs*.

The expression patterns of *GT1* genes were analyzed according to the published transcriptomic data [37]. The *GT1* genes accumulated in all maize tissues, suggesting their contributions to the growth and development of maize. The *GT1* genes were further clustered based on their tissue specificity in gene expression, and divergent expression patterns were found across different phylogenetic groups. It is universal that the *GT1* genes from the same group may have different expression patterns, such as *Zm00001eb051070* and *Zm00001eb146840* in Group A, *Zm00001eb150460* and *Zm00001eb291420* in Group J, and *Zm00001eb056990* and *Zm00001eb158880* in Group Q (Figure 4). The expression patterns also varied among duplicated gene pairs. The duplicated gene pair of *Zm00001eb170440* and *Zm00001eb113190* showed similar expression patterns with the highest expression in mature leaf, whereas the gene pairs *Zm00001eb194070* and *Zm0001eb279380* were highly expressed in pericarp and silk, respectively (Figure 4).

### 3.5. Candidate GT1 Genes in Anthocyanin Biosynthesis

Theoretically, genes involved in the same metabolic pathway can be highly co-expressed in plant tissues [42]. The last two steps of the biosynthetic pathways of anthocyanins are catalyzed by ANS and GT1 (Figure S2). Thus, to identify the GT1 involved in anthocyanin biosynthesis, we conducted gene expression profiling with *ANS* genes. A set of 14 *ANS* genes were identified in maize by searching for ANS orthologs of *Arabidopsis* (Table S4). The results showed that 16 GT1s exhibited similar expression patterns to those of *ANSs* and clustered well with *ANSs* (Figure 4), which might serve as candidate genes contributing to glycosylation in anthocyanin biosynthesis. Of these *GT1s*, only *Zm00001eb374230* has been announced as putative anthocyanidin 3GT involved in anthocyanin biosynthesis [43].

**Figure 5:**
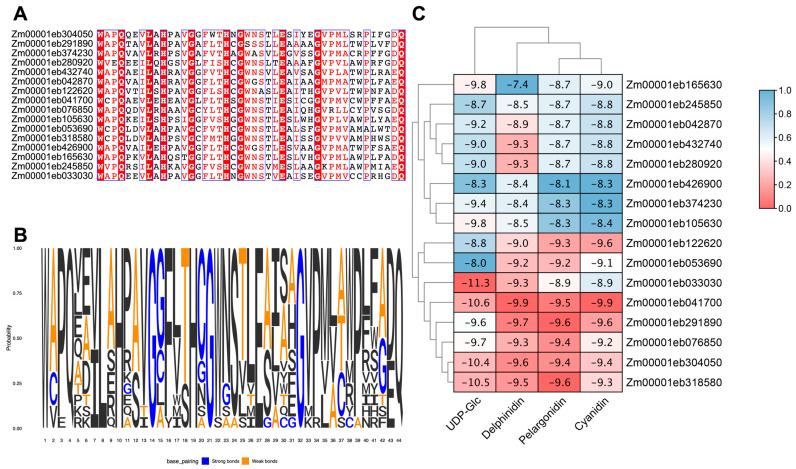
Molecular docking analysis of maize GT1s. (**A**) Multiple sequence alignment of the PSPG motifs from 16 selected GT1 protein that may be involved in anthocyanin biosynthesis. (**B**) The amino acid frequency in the conserved PSPG motifs of GT1 proteins. (**C**) Heat map of docking results for GT1 with different substrates. The numbers indicate affinity energies (kcal/mol). The phylogenetic relationships are shown on the top and left.

These 16 *GT1* candidates belonged to nine phylogenetic groups, including three members in Groups D and E; two members each in Groups H, L, and Q; and one each in Groups C, F, G, and N. Multiple sequence analysis showed that the majority of the residues in the PSPG motif were highly conserved with consistent amino acids in positions 1 (W), 4 (Q), 8 (L), 10 (H), 14 (G), 19 (H), 21 (G), 27 (E), 32 (G), and 44 (Q) (Figure 5A,B). The results suggested that these 16 GT1s might participate in similar biological pathways.

### 3.6. Molecular Docking of GT1s

**Figure 6:**
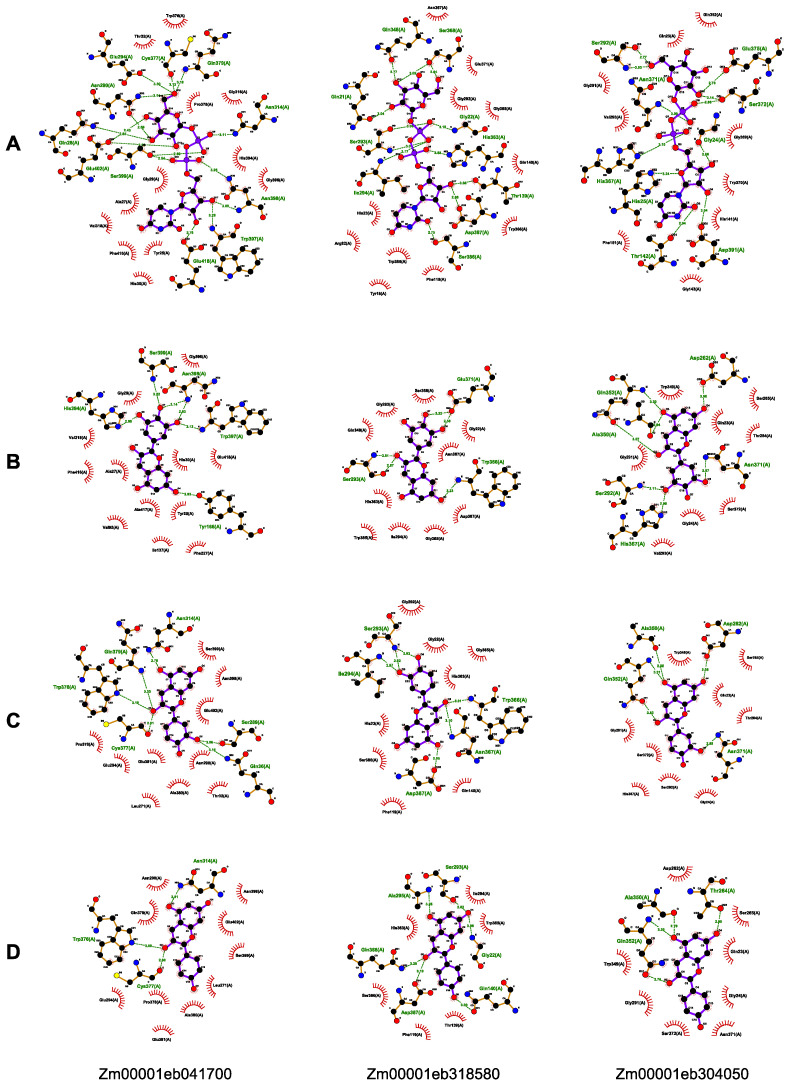
Molecular docking of three selected GT1s with UPD-Glc (**A**), delphinidin (**B**), pelargonidin (**C**), and cyanidin (**D**). The compounds with purple bonds represent the ligands, and the amino acids with brown bonds are from the receptors. Green dotted line between the amino acids represents hydrogen bonding and other amino acids show hydrophobic interactions. The black, red, blue and yellow balls represent the C, O, N and S atoms, respectively.

The molecular docking study was carried out to examine the binding interactions of GT1s with the substrates, UDP-Glc, delphinidin, pelargonidin, and cyanidin, associated with anthocyanin glycosylation. All the 16 GT1s exhibited high affinity with the tested substrates, displaying minimum binding energies ranging from −8.0 to −11.3 kcal/mol with UDP-Glc, −7.4 to −9.9 kcal/mol with delphinidin, −8.1 to −9.6 kcal/mol with pelargonidin, and −8.3 to −9.9 kcal/mol with cyanidin. Zm00001eb033030 demonstrated the strongest binding to UDP-Glc (−11.3 kcal/mol), Zm00001eb041700 bound strongly to delphinidin (−9.9 kcal/mol) and cyanidin (−9.9 kcal/mol), and Zm00001eb291800 and Zm00001eb318580 had a comparable higher binding affinity to pelargonidin (−9.6 kcal/mol). Furthermore, three GT1s, Zm00001eb041700, Zm00001eb304050, and Zm00001eb318500, showed a better docking efficiency with all four substrates (Figure 5C). The molecular docking simulation indicated that GT1s might interact with the four substrates by forming several hydrogen bonds and hydrophobic interactions (Figure 6). UDP-Glc mainly interacted with GT1s around amino acid residues of the PSPG motif (Figure 6A).

## 4. Discussion

GT1 is the largest family of glycosyltransferase. To date, the CAZy database collected over 43,000 proteins of GT1s from nearly 8000 species, including bacteria, animals, plants, fungi, and viruses. Approximately one third of the GT1 members were from plants. A total of 316 GT1s have been identified from different maize lines, and only 107 unigenes were retrieved from the B73 reference genome through sequence blasts of each GT1. Some GT1 proteins from the CAZy database might be aligned to the same reference gene. However, sequence differences in these GT1s might lead to a divergence in substrate recognition and catalyzation.

Phylogenetic analysis revealed that Groups E and L have the most GT1 members, consistent with previous studies in *Arabidopsis*, maize, sorghum, and grape [20] [25]. Group B always contains limited gene members [20]. Group B has a relatively close relationship with Group Q and all the GT1s close to Group B were precisely divided into Group Q, which might be why no GT1 belonging to Group B was identified in this study. Conserved motif analysis showed that the C-terminus of GT1s had more conserved motifs including PSPG motifs, which is related to the recognition and catalyzation of UDP-Glc. Similarly, GT1 had a higher affinity with the donor sugar UDP-Glc but not with the receptor molecules in molecular docking analysis. Interestingly, even if some GT1 genes contained a close phylogenetic relationship and were classified into the same group, they might have different conserved motif distributions and gene structures, leading to the development of new biological functions.

Gene duplication is one of the main forces acting on gene expansion and finally promoting the evolution of organisms [44]. A total of 58 *GT1s* were evolved from either WSD or segmental duplication, or tandem duplication. Both types of gene duplication equally contributed to the expansion of *GT1s*, whereas the *GT1* family might primarily evolve through tandem duplication in *Arabidopsis* [45]. We also found motif duplication in some *GT1* members, which might be another manner to expand *GT1* members with novel functions.

For the *GT1* gene members, we were specifically interested in those that might play roles in anthocyanin biosynthesis. *Zm00001eb374230* (*BZ1*) is predicted to encode a 3GT, and a mutation of *BZ1* resulted in reduced anthocyanin accumulation in the seed aleurone layer, the seeding coleoptile, and the stem of maize plants [43]. Expression profiles and molecular docking analysis suggested that some *GT1s* play an important role in anthocyanin biosynthesis. Three GT1s, Zm00001eb033030, Zm00001eb304050, and Zm00001eb318580, have stronger binding to the donor and receptors than BZ1, indicating their potential functions in the production of higher anthocyanins. This speculation also needs to be further validated through experimental analysis.

In conclusion, a total of 107 *GT1s* were obtained from the whole-genome identification of the reference genome and systemically analyzed. All GT1s were highly conserved, containing the PSPG motif and glycosyltransferase-related domain. Gene duplication and motif duplication expanded *GT1* members at the whole-genome level and provided new gene births during the evolution of maize. Candidate *GT1s* in anthocyanin biosynthesis were predicted through expression analysis with *ANS* and testified through molecular docking. The results are beneficial for the functional study of *GT1s* and will promote the production of anthocyanin biosynthesis in synthetic biology.

## Figures and Tables

**Table 1:** Characteristics of *GT1* genes in maize genome.

Gene ID	Chr	Chromosomal Position	Length (aa)	Gene Symbol	Classification	Number of Introns	Gene Type ^1^
*Zm00001eb020950*	Chr1	79115933–79117889	473		L	0	Proximal
*Zm00001eb020970*	Chr1	79160471–79162703	465		L	2	Tandem
*Zm00001eb020980*	Chr1	79196751–79198788	465		L	2	Tandem
*Zm00001eb033030*	Chr1	184391611–184393452	462	*BX9*	H	1	Dispersed
*Zm00001eb041700*	Chr1	219547437–219549217	496		G	0	Dispersed
*Zm00001eb042870*	Chr1	226122699–226124455	507		D	0	Dispersed
*Zm00001eb047200*	Chr1	242648507–242654443	492		K	1	WGD or Segmental
*Zm00001eb051070*	Chr1	259782541–259784813	516		A	1	Dispersed
*Zm00001eb053690*	Chr1	269552894–269554690	471	*IAGLU1*	L	2	Dispersed
*Zm00001eb056990*	Chr1	282218667–282220404	474		Q	1	Dispersed
*Zm00001eb057740*	Chr1	284753755–284758573	460		I	2	Tandem
*Zm00001eb057750*	Chr1	284764856–284766611	457		I	0	WGD or Segmental
*Zm00001eb057770*	Chr1	284774974–284776823	459		I	1	Tandem
*Zm00001eb057790*	Chr1	284779663–284781743	458		I	1	Tandem
*Zm00001eb061920*	Chr1	298006146–298007982	469		I	1	Dispersed
*Zm00001eb074330*	Chr2	19866206–19867838	467	*CZOG1*	O	0	Proximal
*Zm00001eb074370*	Chr2	20057564–20059103	465		O	0	Proximal
*Zm00001eb076850*	Chr2	28481546–28485790	511	*SK1*	N	1	WGD or Segmental
*Zm00001eb077990*	Chr2	32158225–32160541	471		M	0	Dispersed
*Zm00001eb081210*	Chr2	45506320–45508428	493		G	1	Proximal
*Zm00001eb081230*	Chr2	45701694–45703531	487		G	1	WGD or Segmental
*Zm00001eb085770*	Chr2	76160840–76162497	466		O	0	Dispersed
*Zm00001eb087380*	Chr2	95946783–95948674	489		G	0	Dispersed
*Zm00001eb094890*	Chr2	162271068–162276069	492	*UGT5174*	H	1	WGD or Segmental
*Zm00001eb096940*	Chr2	174552351–174554493	470		H	1	WGD or Segmental
*Zm00001eb098300*	Chr2	180463741–180465530	476		N	1	Dispersed
*Zm00001eb105190*	Chr2	206674564–206676436	480		P	0	Dispersed
*Zm00001eb105580*	Chr2	208529035–208530699	427	*SM2*	A	1	Dispersed
*Zm00001eb105610*	Chr2	208610550–208612222	480		E	0	WGD or Segmental
*Zm00001eb105630*	Chr2	208612412–208613837	470		E	0	WGD or Segmental
*Zm00001eb107750*	Chr2	214236761–214238763	474		F	1	Dispersed
*Zm00001eb107760*	Chr2	214252312–214254275	470		A	1	Dispersed
*Zm00001eb110790*	Chr2	222058692–222060708	456		M	0	Dispersed
*Zm00001eb113190*	Chr2	229706824–229708687	487		D	0	WGD or Segmental
*Zm00001eb114130*	Chr2	232266306–232268281	476		E	0	Dispersed
*Zm00001eb122620*	Chr3	10681696–10683755	525		D	0	WGD or Segmental
*Zm00001eb122630*	Chr3	10830992–10832903	484		D	0	Tandem
*Zm00001eb135190*	Chr3	115806978–115812646	471		E	4	Dispersed
*Zm00001eb138250*	Chr3	137388625–137390462	491		G	0	Dispersed
*Zm00001eb146840*	Chr3	180787206–180789032	476		A	0	Tandem
*Zm00001eb146850*	Chr3	180819236–180820720	473		A	0	Tandem
*Zm00001eb146860*	Chr3	180824047–180826007	512		A	0	Tandem
*Zm00001eb150460*	Chr3	192562189–192565830	474		J	1	WGD or Segmental
*Zm00001eb150470*	Chr3	192567391–192571400	452		J	2	Tandem
*Zm00001eb154000*	Chr3	205428339–205450268	488		E	9	Tandem
*Zm00001eb158880*	Chr3	221304007–221307439	474		Q	0	Dispersed
*Zm00001eb165410*	Chr4	3542471–3544431	490		L	0	Dispersed
*Zm00001eb165590*	Chr4	4060833–4062879	491	*BX8*	H	2	Dispersed
*Zm00001eb165630*	Chr4	4116762–4118560	491		E	0	Dispersed
*Zm00001eb167530*	Chr4	8333244–8335422	502		E	1	Dispersed
*Zm00001eb170440*	Chr4	24818567–24820532	516		D	0	WGD or Segmental
*Zm00001eb170510*	Chr4	25081389–25083426	483		D	0	Proximal
*Zm00001eb172500*	Chr4	34588042–34589834	490		I	1	Dispersed
*Zm00001eb194060*	Chr4	185323500–185325440	484		G	1	Proximal
*Zm00001eb194070*	Chr4	185374820–185376744	484		G	1	WGD or Segmental
*Zm00001eb196550*	Chr4	192838263–192841161	522		L	0	WGD or Segmental
*Zm00001eb196630*	Chr4	193168606–193170298	458	*UFGT4*	A	0	Dispersed
*Zm00001eb204620*	Chr4	236014681–236017119	503	*CEP2*	E	0	Tandem
*Zm00001eb204630*	Chr4	236082248–236084078	485		E	0	Tandem
*Zm00001eb206400*	Chr4	243100226–243107731	500		L	0	WGD or Segmental
*Zm00001eb234750*	Chr5	101616810–101621419	491		L	4	WGD or Segmental
*Zm00001eb240450*	Chr5	161029842–161031557	502		D	0	Tandem
*Zm00001eb240460*	Chr5	161031522–161033144	474		D	0	Tandem
*Zm00001eb244570*	Chr5	178888288–178890479	488		G	1	Dispersed
*Zm00001eb245850*	Chr5	183441650–183444276	462		C	1	Tandem
*Zm00001eb279380*	Chr6	122762448–122764350	470		G	1	WGD or Segmental
*Zm00001eb279510*	Chr6	123180906–123182259	447		D	0	Dispersed
*Zm00001eb280920*	Chr6	130691016–130693844	475	*CGT1*	E	0	WGD or Segmental
*Zm00001eb280930*	Chr6	130704890–130706769	482	*UGT1*	E	0	Tandem
*Zm00001eb280950*	Chr6	130831473–130833292	483	*CGT2*	E	0	Proximal
*Zm00001eb290970*	Chr6	166328839–166330747	491		G	0	Dispersed
*Zm00001eb291420*	Chr6	167093344–167095148	457		J	1	WGD or Segmental
*Zm00001eb291880*	Chr6	168153151–168154910	509		Q	0	WGD or Segmental
*Zm00001eb291890*	Chr6	168199823–168201808	493		Q	0	Tandem
*Zm00001eb293450*	Chr6	171371429–171373586	473		E	0	Tandem
*Zm00001eb293460*	Chr6	171373527–171375229	478		E	0	WGD or Segmental
*Zm00001eb294040*	Chr6	172699717–172701409	482		M	0	Dispersed
*Zm00001eb304010*	Chr7	23801316–23803037	463	*UGT9250*	H	1	WGD or Segmental
*Zm00001eb304050*	Chr7	24029052–24030857	469	*UFGT2*	H	1	Tandem
*Zm00001eb304060*	Chr7	24154958–24156803	482		H	1	Proximal
*Zm00001eb304070*	Chr7	24181111–24183054	466		H	1	Proximal
*Zm00001eb304120*	Chr7	24588945–24591016	468	*CNGT1*	H	1	Proximal
*Zm00001eb318570*	Chr7	146013215–146015100	472		L	1	WGD or Segmental
*Zm00001eb318580*	Chr7	146096773–146098587	470		L	1	Tandem
*Zm00001eb318970*	Chr7	147684571–147686411	456		G	1	Dispersed
*Zm00001eb322260*	Chr7	160217163–160219108	476		E	0	Tandem
*Zm00001eb322270*	Chr7	160220531–160222324	484		E	0	Tandem
*Zm00001eb323960*	Chr7	165932228–165934061	483		E	0	Tandem
*Zm00001eb323970*	Chr7	166034566–166036489	518	*UMC2716*	E	0	Tandem
*Zm00001eb330430*	Chr7	181720552–181722435	480		A	0	WGD or Segmental
*Zm00001eb337060*	Chr8	19469860–19471775	500		L	0	WGD or Segmental
*Zm00001eb337300*	Chr8	20579174–20581428	476		L	2	Dispersed
*Zm00001eb343000*	Chr8	67212322–67213925	465		L	0	WGD or Segmental
*Zm00001eb353640*	Chr8	128038314–128040087	465		E	0	WGD or Segmental
*Zm00001eb360890*	Chr8	156736301–156738932	480		E	0	Dispersed
*Zm00001eb367250*	Chr8	173936706–173940214	463	*CZOG2*	O	0	Dispersed
*Zm00001eb371300*	Chr8	182117851–182119448	468		E	0	Tandem
*Zm00001eb371350*	Chr8	182219335–182220996	477		E	0	WGD or Segmental
*Zm00001eb374230*	Chr9	13120306–13122164	471	*BZ1*	F	1	Dispersed
*Zm00001eb380880*	Chr9	41151938–41153717	479		E	0	WGD or Segmental
*Zm00001eb381410*	Chr9	46177110–46178902	484		E	0	Dispersed
*Zm00001eb386000*	Chr9	92897492–92899854	472	*UMC2700*	L	0	Tandem
*Zm00001eb394980*	Chr9	136330935–136332755	486		E	0	Dispersed
*Zm00001eb419710*	Chr10	102460083–102462158	474		L	2	Tandem
*Zm00001eb419720*	Chr10	102642521–102644377	484		L	2	Tandem
*Zm00001eb426900*	Chr10	134433367–134435275	500		D	0	Dispersed
*Zm00001eb432740*	Chr10	148886364–148888273	482		Q	0	Dispersed

^1^ Gene type was determined through collinearity analysis. Dispersed means that the gene might arise from transposition. Proximal means that the gene might arise from small-scale transposition or arise from tandem duplication and insertion of some other genes. WGD or segmental means that the gene might arise from whole-genome duplication (WSD) or segmental duplication.

## Data Availability

Data are contained within the article.

## Supplementary Materials

The following supporting information can be downloaded at https://www.mdpi.com/article/10.3390/genes14112099/s1, Figure S1: Multiple sequence alignment of 107 GT1 proteins; Figure S2: Expression profiles of *ANS* genes involved in anthocyanin biosynthesis; Table S1: Conserved motif features of GT1s; Table S2: Summary of gene clusters of GT1s in all maize chromosomes; Table S3: Summary of duplicated gene pairs of GT1s; Table S4: Putative *ANS* genes of maize.
