# Supplementary material for: Characterization of Glycosyltransferase Family 1 (GT1) and Their Potential Roles in Anthocyanin Biosynthesis in Maize
Source: Genes (Basel). 2023 Nov 18;14(11):2099. doi: 10.3390/genes14112099 (PMC10671782; doi:10.3390/genes14112099)
Supplement: Supplementary file 1 [file genes-14-02099-s001.zip › Supplementary_Figures.pdf]

Figure S1

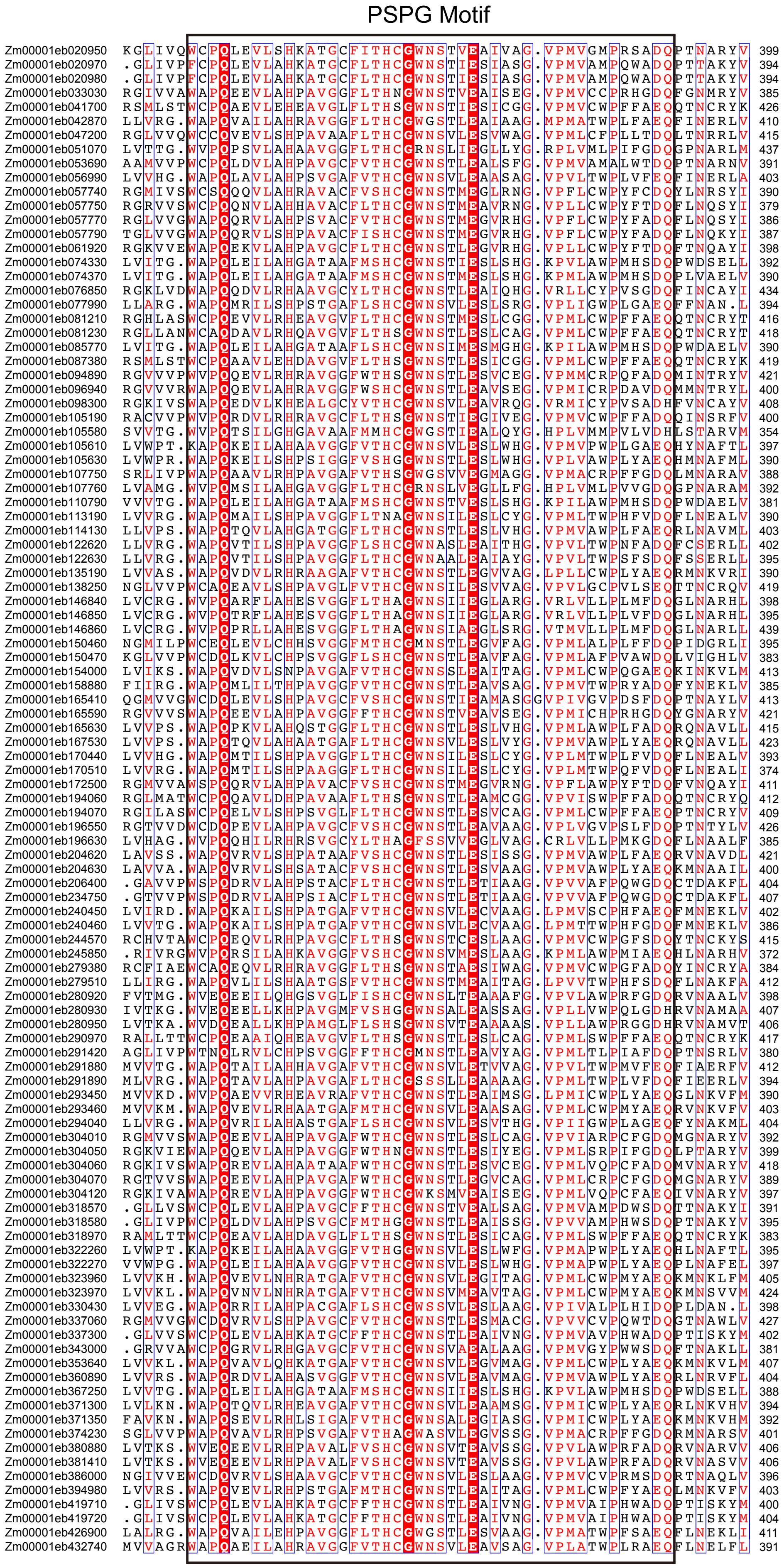

Figure S1 Multiple sequence alignment of 107 GT1 proteins. All GT1 proteins contain the conserved PSPG motif (black box) in their C-terminus.

Figure S2

A

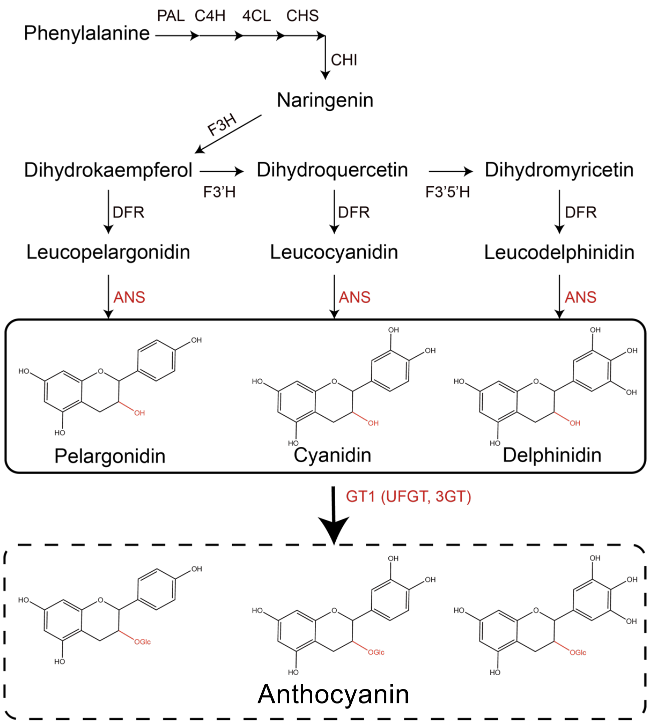

B

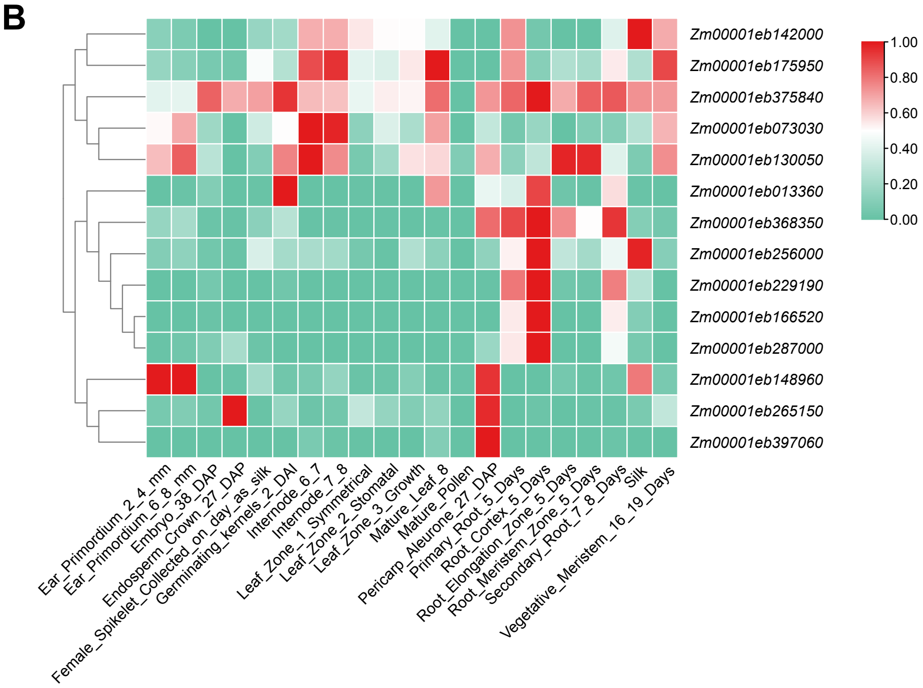

Figure S2 Expression profiles of ANS genes involved in anthocyanin biosynthesis. **A** The pathway of anthocyanin biosynthesis. PAL, phenylalanine ammonia lyase; C4H, cinnamate 4-hydroxylase; 4CL, 4-coumarate CoA ligase; CHS, chalcone synthase; CHI, chalcone isomerase; F3H, flavanone 3-hydroxylase; F3′H, flavonoid 30 hydroxylase; F3′5′H, flavonoid 3050hydroxylase; DFR, dihydroflavonol 4-reductase; ANS, anthocyanidin synthase; 3GT, UDP-galactose flavonoid 3-O-galactosyltransferase. B Heat map of ANS gene expression in multiples maize plant tissues. The relative expression levels are depicted according to the color scale, where a change from green to red indicates transcript abundance from low to high. The phylogenetic relationships are shown on the left.
