# Supplementary material for: Characterization of Glycosyltransferase Family 1 (GT1) and Their Potential Roles in Anthocyanin Biosynthesis in Maize
Source: Genes (Basel). 2023 Nov 18;14(11):2099. doi: 10.3390/genes14112099 (PMC10671782; doi:10.3390/genes14112099)
Supplement: Supplementary file 1 [file genes-14-02099-s001.zip › Supplementary_Tables.pdf]

Table S1 Conserved motif features of GT1s.

Motif No.	Logo	E-value	Sites	Width	Motif Sequence
1	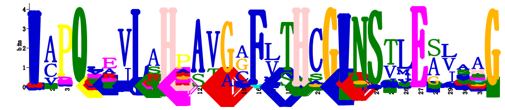	1.7e-2417	107	32	WAPQLEVLAHPAVGAFLTHCGWNSTLEALAAG
2	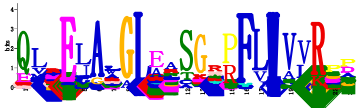	1.6e-1116	105	23	QLRELALGLEASGRPFLWVVRPP
3	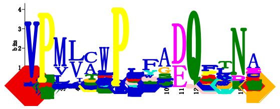	2.1e-801	105	16	VPMLCWPLFADQFTNA
4	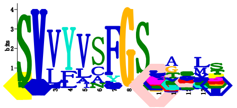	1.1e-611	105	14	SVVYVSFGSLASLS
5	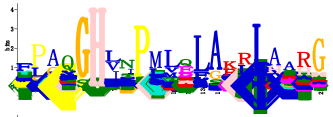	3.5e-765	105	21	FPAQGHLNPMLQLAKRLAARG
6	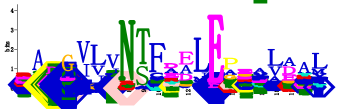	4.5e-565	104	21	RADGVLVNTFAELEPEALAAL
7	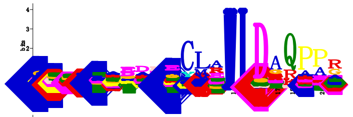	4.7e-512	105	21	GSSLWAEDHECLAWLDAQPPR
8	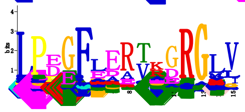	2.3e-354	83	15	LPEGFLERTKGRGLV
9	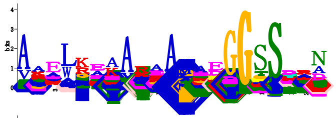	9.1e-405	90	21	AAELKEAARAAVAEGGSSDRN
10	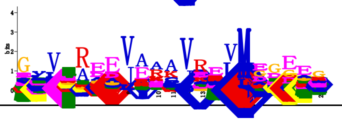	3.1e-383	102	21	GVVTREEVAAAVREVMEGEEG

Table S2 Summary of gene clusters of GT1s in all maize chromosomes.

No.	Chr	Clustered Genes
1	Chr1	*Zm00001eb020950*, *Zm00001eb020970*, *Zm00001eb020980*
2	Chr1	*Zm00001eb057740*, *Zm00001eb057750*, *Zm00001eb057770*, *Zm00001eb057790*
3	Chr2	*Zm00001eb074330*, *Zm00001eb074370*
4	Chr2	*Zm00001eb081210*, *Zm00001eb081230*
5	Chr2	*Zm00001eb105580*, *Zm00001eb105610*, *Zm00001eb105630*
6	Chr2	*Zm00001eb107750*, *Zm00001eb107760*
7	Chr3	*Zm00001eb122620*, *Zm00001eb122630*
8	Chr3	*Zm00001eb146840*, *Zm00001eb146850*, *Zm00001eb146860*
9	Chr3	*Zm00001eb150460*, *Zm00001eb150470*
10	Chr4	*Zm00001eb165410*, *Zm00001eb165590*, *Zm00001eb165630*
11	Chr4	*Zm00001eb170440*, *Zm00001eb170510*
12	Chr4	*Zm00001eb194060*, *Zm00001eb194070*
13	Chr4	*Zm00001eb196550*, *Zm00001eb196630*
14	Chr4	*Zm00001eb204620*, *Zm00001eb204630*
15	Chr5	*Zm00001eb240450*, *Zm00001eb240460*
16	Chr6	*Zm00001eb279380*, *Zm00001eb279510*
17	Chr6	*Zm00001eb280920*, *Zm00001eb280930*, *Zm00001eb280950*
18	Chr6	*Zm00001eb291880*, *Zm00001eb291890*
19	Chr6	*Zm00001eb293450*, *Zm00001eb293460*
20	Chr7	*Zm00001eb304010*, *Zm00001eb304050*, *Zm00001eb304060*, *Zm00001eb304070*, *Zm00001eb304120*
21	Chr7	*Zm00001eb318570*, *Zm00001eb318580*
22	Chr7	*Zm00001eb322260*, *Zm00001eb322270*
23	Chr7	*Zm00001eb323960*, *Zm00001eb323970*
24	Chr8	*Zm00001eb371300*, *Zm00001eb371350*
25	Chr10	*Zm00001eb419710*, *Zm00001eb419720*

Table S3 Summary of duplicated gene pairs of GT1s.

No.	Duplicated Gene	
1	*Zm00001eb063070*	*Zm00001eb096940*
2	*Zm00001eb047200*	*Zm00001eb222590*
3	*Zm00001eb057750*	*Zm00001eb214570*
4	*Zm00001eb063070*	*Zm00001eb304010*
5	*Zm00001eb113190*	*Zm00001eb170440*
6	*Zm00001eb081230*	*Zm00001eb244710*
7	*Zm00001eb076850*	*Zm00001eb248110*
8	*Zm00001eb105610*	*Zm00001eb322190*
9	*Zm00001eb105630*	*Zm00001eb322210*
10	*Zm00001eb111270*	*Zm00001eb330430*
11	*Zm00001eb102880*	*Zm00001eb318570*
12	*Zm00001eb096940*	*Zm00001eb304010*
13	*Zm00001eb094890*	*Zm00001eb304010*
14	*Zm00001eb163360*	*Zm00001eb196550*
15	*Zm00001eb150460*	*Zm00001eb291420*
16	*Zm00001eb122620*	*Zm00001eb283060*
17	*Zm00001eb156460*	*Zm00001eb343000*
18	*Zm00001eb153990*	*Zm00001eb353640*
19	*Zm00001eb122240*	*Zm00001eb337060*
20	*Zm00001eb194070*	*Zm00001eb279380*
21	*Zm00001eb206400*	*Zm00001eb234750*
22	*Zm00001eb291880*	*Zm00001eb352700*
23	*Zm00001eb293440*	*Zm00001eb371350*
24	*Zm00001eb293460*	*Zm00001eb371290*
25	*Zm00001eb280920*	*Zm00001eb380880*

Table S4 Putative *ANS* genes of maize.

Gene ID	Chr	Chromosomal Position	Gene Symbol
Zm00001eb013360	Chr1	44729549–44735265	
Zm00001eb073030	Chr2	15774137–15777049	
Zm00001eb130050	Chr3	52947715–52950003	*SRG1*
Zm00001eb142000	Chr3	156270441–156272338	
Zm00001eb148960	Chr3	187946425–187951771	
Zm00001eb166520	Chr4	5991734–5992330	
Zm00001eb175950	Chr4	53715253–53717388	
Zm00001eb229190	Chr5	68086545–68088407	
Zm00001eb256000	Chr5	218475557–218477256	
Zm00001eb265150	Chr6	34319837–34324529	
Zm00001eb287000	Chr6	153979278–153985618	
Zm00001eb368350	Chr8	176088819–176092948	
Zm00001eb397060	Chr9	144069882–144073514	
